# Vitality at home: a phenomenological study of tele-exercise in women aged 80 and older

**DOI:** 10.1186/s11556-024-00360-9

**Published:** 2024-09-19

**Authors:** Janet Lok Chun Lee, Karly Oi Wan Chan, Rick Yiu Cho Kwan, Arnold Yu Lok Wong

**Affiliations:** 1 Department of Rehabilitation Sciences, The Hong Kong Polytechnic University, Hong Kong SAR, China; 2 College of Professional and Continuing Education, The Hong Kong Polytechnic University, PolyU Hung Hom Bay Campus, 8 Hung Lok Road, Hung Hom, Kowloon, Hong Kong SAR, China; 3 School of Nursing, Tung Wah College, Hong Kong SAR, China; 4 Research Institute for Smart Ageing, The Hong Kong Polytechnic University, Hong Kong SAR, China; 5 Research Institute for Sports Science and Technology, The Hong Kong Polytechnic University, Hong Kong SAR, China; 6 Research Centre for Assistive Technology, The Hong Kong Polytechnic University, Hong Kong SAR, China

**Keywords:** Tele-exercise, Oldest-old, Experience, Remotely-delivered home-based exercise, Qualitative, Tele-health

## Abstract

**Background:**

Since the onset of coronavirus 2019, there has been an upsurge of tele-exercise delivery. Previous studies showed old adults find tele-exercise feasible and acceptable. However, there is limited understanding of the oldest-old’s experiences.

**Method:**

This study used the interpretative phenomenological approach. Two semi-structured interviews and home visits were conducted with six oldest-old women, aged between 81 and 91 years, who participated in tele-exercise classes.

**Results:**

Four superordinate themes were identified: ambivalent perception of safety, ease in regular participation, reminded and guided to move the aged body, and technological adaptation.

**Conclusion:**

Our findings indicate that tele-exercise has the potential to assist the oldest-old living in the community in maintaining an adequate activity levels at home, which they perceive as the safest place. Emerging themes provide insights into their lived experiences, enabling service providers to enhance tele-exercise services for this group in the tele-health era.

**Supplementary Information:**

The online version contains supplementary material available at 10.1186/s11556-024-00360-9.

## Background

It has been well recognized that physical activity (PA) has significant health benefits for cardiorespiratory, physical and psychological health [48]. For older adults, maintaining an adequate daily level of PA positively impacts their daily well-being, the aging process, and overall quality of life [47]. Globally, recent statistics showed that the population of the ‘oldest-old’ will be more than tripled between 2015 and 2050, growing from 126.5 million to 446.6 million [24]. Research showed that increased participation in leisure activities was associated with improved quality of life among the oldest-old, and the association was particularly strong among oldest-old who became widowed, developed functional impairments, and had relatively low contact with family [33]. Research has also demonstrated that PA is a protective factor against cognitive impairment, psychological well-being and sarcopenia among the oldest-old adults [6, 17, 20, 45]. However, a systematic review found that over half of the studied oldest-old had low PA levels, and had weak physical status [11], highlighting the importance, of encouraging regular PA among this age group from a public health perspective.

A systematic review [4] investigating factors that encourage and hinder PA participation among community-dwelling oldest-old individuals revealed that home-based exercise programmes and frequent exercise classes (e.g., three times a week) facilitate PA participation. However, factors such as the complexity of exercise, weather conditions, perceived safety (both environmental and personal) and transportation consistently emerged as key barriers to their participation in PA.

According to reviews, exercise interventions for the oldest-old adults were primarily delivered in physical settings with supervision, and some incorporated a mixed-mode approach that included a home practice component supported by home visits or telephone calls by community volunteers or healthcare professional [8, 19]. Recent literature highlights the effectiveness of home-based exercise intervention supported by technologies (e.g., mobile apps and video calls) for the oldest-old. Tiecker et al. [43] found that oldest-old participants rated the intervention as increasingly acceptable over a 12-week period.

Over the last decade, the application of technologies in exercise interventions has evolved and expanded significantly. Such technologies encompass websites, tablet app, mobile app, video-conferencing, and exergaming [7, 25]. Among different types of technology-supported exercise interventions, the definition of tele-exercise is not consistent across the literature [7, 13], this study adopts the definition of tele-exercise as the real-time remote delivery of exercise intervention using videoconferencing software such as Zoom, Skype, Facetime or VooV, along with hardware such as smartphone, tablet, or computer with a web camera, and network connections [49]. Unlike web-based or mobile app-based exercise interventions, and exergaming, tele-exercise enables synchronous two-way video and audio transmission, allowing both the instructor and the participants to see, hear and interact with each other in real-time, similar to exercise intervention delivered in physical settings.

Recent research has provided preliminary evidence that tele-exercise can provide health benefits similar to face-to-face delivery. For instance, a study demonstrated an improvements in depressive symptoms and blood pressure among older adults after tele-exercise [2]. Another study also revealed that tele-exercise interventions can promote muscle strength, balance and flexibility in older adults [1]. Moreover, tele-exercise has been well accepted by older adults, as reflected in their satisfactory compliance rates [38, 49]. A Brazilian study showed the feasibility of implementing tele-exercise among the older adults, with no reported adverse event during the implementation period [9].

To date, several feasibility and qualitative studies have examined how the older adults, mainly young-old and middle-old perceived and experienced this new mode of exercise delivery. They found that tele-exercise addressed their environmental barriers, such as transportation and accessibility [9, 23, 26, 49]. Additionally, older adults valued the opportunity to learn new things, including using technology for communication and exercise [14]. However, they report some negative perceptions of tele-exercise, such as technical barriers and compromised supervision quality [21].

While previous studies offered insights into older adults’ general experiences and perspectives regarding tele-exercise, these studies mainly reflected the views and experiences of older adults across broad age range (e.g., 55 to 89 years), with majority in their 60 s and 70 s [21]. There is limited in-depth understanding of how the oldest-old adults perceive and experience tele-exercise. Studying their tele-exercise experience is crucial, as this group has a stronger demand for home-based PA intervention than their younger counterparts because of challenges with transportation and reliance on family or caregivers [8]. While home-based tele-exercise has the potential to be appealing to the oldest old, research showed that the oldest-old have lower eHealth literacy than their younger counterparts [18]. There is a need to study the experience of this group in greater depth. To this end, the interpretative phenomenological approach (IPA) may help deepen the understanding of the adoption of tele-exercise services among the oldest-old. The IPA involves an in-depth understanding and explanation of how participants make sense of their own world by zooming in on particular experiences [34]. It uses a phenomenological lens to uncover the nuanced and contextualized tele-exercise experiences. By adopting this theoretical orientation, the data collection and analysis conducted by researchers will allow the phenomenon itself to reveal its essence. This approach enables the emergence of novel, interesting, and unexpected phenomena. Additionally, IPA has been effectively used to understand health and exercise experiences in previous studies [35].

During the COVID-19 in which social distancing policy was in place, all exercise classes which normally conducted in community centres were changed to be delivered online [21]. After the pandemic, even though it was not necessary to offer tele-exercise classes, some senior community centres still offered tele-exercise as community support services. They received funding support from the government to procure and rent technology products to improve the quality of life of older adults [42]. This study explores the presupposing ideas about lived experience of tele-exercise among the oldest-old in detail. For instance, how the oldest-old with lower level of technological literacy manage to participate in the remotely-delivered tele-exercise class? In previous literature on oldest-old adults, different age cut-offs were used to define this group; the current study defined the term “oldest-old” as individuals aged 80 years or older [44]. This study aimed to describe the lived experience of tele-exercise among community-dwelling older adults aged 80 years or above; and to provide an in-depth analysis of their experience in order to enhance the implementation of tele-exercise services for this subgroup in the future.

This study presents the lived experience of community-living individuals aged 80 years or older in Hong Kong, serving as a source for further learning of how they perceive and experience tele-exercise, as well as how they manage their participation. This was achieved through repeated interviews and observations of their experiences during home visits.

## Methods

### Design

This was a naturalistic study informed by IPA [36]. IPA was chosen as the theoretical underpinning as this study aimed to generate in-depth understanding beyond what could be found in feasibility studies or descriptive qualitative research. It enables researchers to investigate complex phenomena and allows for in-depth exploration and understanding of individuals’ lived experiences and subjective perspectives. This study was reported according to the Consolidated criteria for reporting qualitative research (COREQ).

### Sampling

Purposive sampling was adopted as a sampling strategy. Recruitment took place between December 2022 to March 2023. Recruitment emails were sent to territory-wide older adults’ community centres, commercial fitness centres, fitness association, and the research network of the first author in Hong Kong, S.A.R. in December 2022. Community-dwelling older adults, aged 80 years or above, with experience in tele-exercise were invited to participate in this study. The inclusion criteria were: a) community-dwelling older adults aged 80 years or above; and b) having at least three months of tele-exercise experience and currently participating in tele-exercise. As this study aimed to delve into the lived tele-exercise experience of oldest-old adults through observations in a home setting, and discover findings that could not be acquired through verbal interviews, those who had prior tele-exercise experience who had since discontinued participation, and could only tell their experience by recalling their memory were excluded. In addition, those with low cognitive and verbal abilities that prevented engagement in research conversation, were excluded.

### Sample size consideration

Considering the presence of social factors and psychological variability that may cause patterns of convergence and divergence within a group, IPA prefers smaller sample sizes to ensure homogeneity within a group. This approach recognizes that a detailed and interpretative account can only be realistically achieved with small samples [34]. Previous research using IPA considered six participants as adequate sample size [3].

### Participants

#### Demographic and other characteristics

Age, gender, housing type, living arrangement, and perceived health status were collected. Perceived health status was indicated by responding to categorical responses, namely: very poor, bad, fair, good and excellent. Technological acceptance was measured using the Senior Technology Acceptance Model 14-item scale (STAM-14). STAM-14 comprises 14 items, with each item rated on a 10-point scale from 1 being very unsatisfied to 10 being very satisfied. STAM-14 consists of four constructs: attitudinal beliefs, control beliefs, gerontechnological anxiety, and health. The total scores range from 14 to 140, with a higher score indicating a higher level of acceptance towards technology by older adults. The scale has been shown to have good internal consistency (Cronbach’s α = 0.817 − 0.915) and construct validity (AVE = 0.155 − 0.795) [5]. These characteristics were collected as research showed that living environment, social environment, functional health and technology interest were the key characteristics that influenced technology adoption and experience [30].

Six community-dwelling women, who were members of four different district-based senior community centres, were successfully recruited and interviewed (6 women; mean age: 86 years, range: 81–92 years). It is worth to note that this study did not intend to exclude men. The recruitment of female participants only may be attributed to the fact that the average life expectancy of 82.5 years old. There are significantly more women than men aged 80 years or above [37].

#### Ethics and consent

This study obtained ethics approval from the Institutional Review Board of The Hong Kong Polytechnic University (approval number: HSEARS20221129001). Information sheets with details of the study and issues regarding voluntary participation, confidentiality and potential risks, and benefits were provided and explained to the participants before the commencement of data collection, and written consent was obtained.

### Procedure

#### Interviews

Interviews were carried out between December 2022 and March 2023, with each participant being interviewed twice. Participants joined the interview as they were informed that a university was conducting research that investigate oldest-old tele-exercise experience. A relationship between interviewer and interviewee was not established before the commencement of the study. The first interview was conducted through Zoom and the second interview was conducted during home visit. First and second interviews were two to three weeks apart, allowing adequate time for preliminary data analysis. The average interview duration combining first and second interview was 40 min, minimum was 34 min and maximum was 55 min. All interviews were conducted by the first author. The first author is an experienced female qualitative researcher with a PhD qualification, and have seven years of experience in conducting exercise and illness experience qualitative research. Open-ended and conversational style questions were used to explore various aspects of their tele-exercise experiences. The following interview frame was used: oldest-old’s perception of the class, the strategies that they employed, any particularly positive or negative aspects in their experiences, and the personal meaning of taking tele-exercise to them. The interviews were conducted in an informal manner, framed as conversations, to elicit natural accounts of the lived tele-exercise experience. The questions served as guidance for participants to tell their lived experience rather than dictating the course of the interview. Participants were encouraged to create the content of the conversation and encouraged to talk as board as possible. Some questions were repeated in each interview to gain a more thorough understanding of their tele-exercise experiences. The first author also discussed findings and clarifying findings in the second interview. This approach allowed for validation of previous statements and identification of recurring patterns.

### Home visit

Being in the setting with participants helps understand their experience and how participants engage in the world [41]. In the current study, the first author visited participants’ homes and observed how they managed the interactive tele-exercise classes. The first author asked participants to show how they participated in the tele-exercise classes and allowed the researcher to observe and video record how they participated in the tele-exercise class. During the video recording, the first author also took notes on participants’ tele-exercise experience. Tele-exercise observations during home visits lasted 40 to 60 min, depending on the class duration. Video clips of each visit were analyzed alongside the transcript. The video clips served as contextual data to support the analysis of interview responses.

### Data saturation

All participants completed two interviews; the second interview ensured all the data within an individual (a case) were collected. As data saturation was achieved in the second interview, a third interview was not arranged. Data saturation within a case is a common strategy applied in IPA studies [29].

## Data analysis

The interviews were transcribed verbatim, and the analysis followed the IPA procedure outlined by Smith [36], consisting of three methodological steps. In the first stage, the analysis started with a case-by-case approach, in which the first author thoroughly examined the transcript of one interview before moving on to the others. For each case, the analysis started by listing all the emergent themes chronologically, based on their appearance in the transcript. In the second stage, the first author made sense of the connections between themes and clustered some of them to form superordinate themes. Certain themes within the transcript that did not align well with the research aim were omitted. After the analysis of the first case, the first author continued to analyze the second case using the same procedure. With the second case, the researcher searched for responses that further articulated the extant themes and identified new and distinct themes in the transcript. Once all cases were analyzed, superordinate themes that emerged across cases were examined. Following all the procedures, there were 13 subordinate themes that encompassed all the transcripts, and from these, four superordinate themes emerged. Each theme, along with quotations from transcripts that provided support, was collated into a table.

### Credibility, transferability, dependability, and confirmability

Trustworthiness was considered in the study through ensuing credibility, transferability, dependability and confirmability [22]. Credibility was ensured through data and analysts triangulation. Throughout all the IPA analytical stages, the understanding of themes was cross-examined with video recordings taken during home visits. In addition, three additional independent researchers with backgrounds in health sciences reviewed the theme tables, and video recording of each case. They tracked the development of each superordinate and subordinate theme and commented on the validity of each theme. The triangulations kept the interpretations in check and facilitated the production of solid evidence. Transferability was ensured through collecting adequate and appropriate demographic characteristics and contextual characteristics of each participant, ensuring the findings of the study can be applied to contexts beyond the original research. Dependability and confirmability were ensured through keeping an audit trail of analysis from initial case by case analyses, to across cases analyses. The keeping of audio-recorded interview content, video-recorded content and verbatim transcriptions also ensured dependability and confirmability.

## Results

**Fig. 1:**
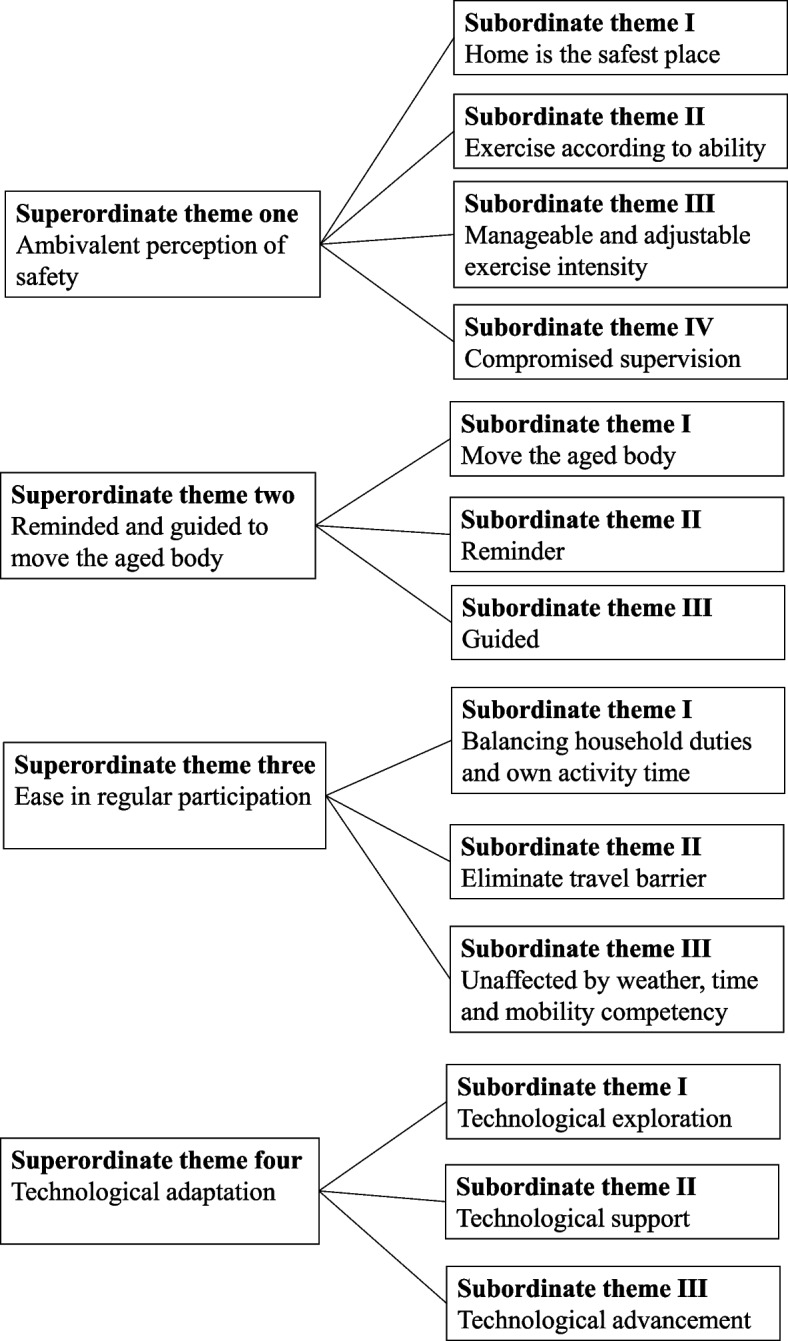
Outline of superordinate and subordinate themes developed from interpretative phenomenological analysis

Four main superordinate themes emerged as the essence of the lived experience of tele-exercise among the oldest-old women (Fig 1).

### Ambivalent perception of safety

The superordinate theme “ambivalent perception of safety” was supported by four subordinate themes: “home is the safest place”, “manageable and adjustable exercise intensity”, “compromised supervision” and “exercise according to ability”.

The oldest-old had ambivalent feelings on the safety of tele-exercise participation. Participant Siu Fong was aware of the lack of immediate and specific supervision and adjustment from the instructor, as reflected by her statement:

“…if there’s anything, he (the instructor) will tell us immediately. Now on zoom, to be honest, we follow (his instructions), if it’s not correct... not correct, um my hands’ position isn’t correct, he can’t tell us how to do it.”

Mei Ling shared similar thoughts:

“Well, I think of course it's good in the classroom. (In) face to face (mode), if there is incorrect (movement), I can listen to you (the instructor). He spoke clearly. On the Internet, there was a burst of sound "ChaChaCha", (the sound is) not clear. In face to face mode, I can hear (the feedback) clearly, and (the instructor can) easily communicate (the feedback). In this sense, face to face mode is better.”

Although supervision might be compromised in tele-exercise, participant perceived the experience of tele-exercise as safe. They highlighted that they felt safe in their home. It was their own “territory”, where they were so familiar with the setting:

“Of course, it feels safer to be at your own place, without illness, you should be safe. They (instructors) can see us, can see us exercising, what are we doing, they're able to see, so we're not worried at all. If you stay at home, you don’t have to wear a mask. In fact, it’s really difficult when the weather’s hot. So, it’s the best to stay home, lots of people like it (tele-exercise at home).” (Siu Ling).

Yuet Ho even asked rhetorically, “How come I’d say it’s safe? Of course it’s the safest to stay at home, isn’t it?”.

Apart from the perceived safeness from the familiar home environment, participants perceived tele-exercise to be safe as they can exercise according to their own ability.

Mui Mui mentioned the instructor would teach them how to make adjustment to exercise movement according to their own ability:

“I can do 4 (repetitions) or 10 (repetitions). But I have to hold a chair to walk. Mr Au (the instructor) taught us, if you feel unstable, hold the chair to support.”

Another participant, Yuet Ho, explained that participant can make own judgement and act according to own ability:

“If you can do it, you follow those movements like others, but if you can’t, I rely on myself and only follow a little bit, it’s also okay…You don’t have to follow all the steps completely. If you… if you feel tired you can rest awhile, no one will scold at you, just take a break, and start again.”

Similarly, Siu Ling also explained that she made her own judgement and avoided those tele-exercise class that was not suitable to her health condition:

“From lying to sitting, um I feel a bit dizzy, so I didn’t attend that class, I told Mr Choy (instructor), he said ‘OK OK OK, don’t come today’. Like when I lie on the ground, and I get up, I feel a bit dizzy, that’s why I didn’t go to that (tele-exercise) class.”

Participants also appreciated the manageable exercise intensity of tele-exercise classes:

“Now on zoom, for us, um… follow him and do it slowly, very good, follow him (instructor) is good, I think he instructs really well, I really have to be slow! People in my age isn’t really fit, can’t be too fast. So, it’s good! Like those aerobic exercise… aerobic exercise, very good. So, it certainly targets those heart and lung function. Those aerobic exercise, those are good. Watched the "TV", follow him doing slowly.” (Siu Fong).

As observed by the researcher, participants were all able to use nearby furniture at home to support exercise movements and participants with weaker physique were seated in most part of the exercise session. They were exercising at an intensity that was manageable to them.

### Reminded and guided to move the aged body

The superordinate theme “reminded and guided to move the aged body” was supported by three subordinate themes, “move the aged body”, “reminder”, and “guided”.

Oldest-old participants acknowledged the importance of “move” or “movement” to avoid stiffness of the body at very old age:

“Now that we’re old, people at this age have to move. Moving is better than not moving, you will feel stiff if you don’t move… If you don’t mind, I’m so old and making mistakes, I will keep doing (tele-exercise). It is pathetic if you can no longer walk.” (Mui Mui).

Yuet Ho understood the importance of exercising her legs:

“For my legs, because I am heavy, I need to better use my legs. I cannot straighten (perform heel raise exercise) my legs for long, other people can straighten it for a few minutes, but I cannot. It keeps shaking. (I feel that) my legs become better after I straighten (perform heel raise exercise) it, it is good for the muscles. For single leg stance, it is good for the muscles, the legs will have strength. With the strengths, you can stand longer, very good. If you do not have the chance (to do exercise), you will fear for fall when you go out, you will also fear that you cannot walk a long distance.”

While it was important to “move”, oldest-old participant appreciated that tele-exercise remind them to ‘move’, as they received reminder message from their phones:

“For “online” (tele-exercise), when it is about time (right before the class starts), the phone will receive a reminder (message), so I will remember. If you don’t remind me, I forget sometimes.” (Siu Ling).

They also appreciated there was no need to remember the exercise by themselves. The exercise steps were being reminded by the instructor by watching the “monitor”:

“For this (type of) exercise, it's my first-time doing exercise facing a “mon” (monitor). Occasionally I also move a bit, I think (this exercise mode) is good, very clear. It’s even better doing exercise at home facing the “mon” (monitor), it is true. Because… mainly it’s because I'm forgetful, you may think I sound clear verbally, but I forget things easily.” (Mui Mui).

Mee Yee appreciated being guided to do exercise by professionals:

“I feel happy, someone is teaching me in this exercise….”

Mui Mui appreciated the knowledge come from the guidance by professionals:

“For practicing Tai Chi in parks, no one is guiding us. We just practice (Tai Chi) by ourselves. Now there is an instructor like Mr.Au, guiding us how to exercise our legs. (Now we know) there is no effect if the knee is facing the floor. Now we have the concept, we are (feel) clearer, isn’t that true?”

The researcher observed that participants responded well to the instructor’s guidance and instructions, and the interactions were similar to in-person class. With the quiet environment at home, the instructor’s voice was the only voice in the environment, making the leadership from instructor strong and positively influenced participants. It was observed that participants had high concentration during the tele-exercise class.

### Ease in regular participation

This superordinate theme is supported by three subordinate themes, “balancing household duties and own activity time”, “eliminate travel barrier”, and “unaffected by weather, time and mobility competency”.

Yuet Ho appreciated the convenience of participating tele-exercise. They could manage household duties with their own activity time:

“(tele-exercise) is good, it is much more convenient. I don’t have to rush to the community centre, I can (turn on) computer first and do other stuff.”

Oldest-old participants mentioned that it was not easy for them to leave their homes and travel somewhere for exercise. The oldest-old women appreciated that tele-exercises eliminated the travel barrier and the experience was unaffected by weather and mobility issues:

“On one hand, I am old now, I have to walk on my own… The road here, the road is steep, I have to carry my walking stick, sometimes… I am afraid of falling, so I seldom go out. From my home to the community centre, it takes at least 20 min if I walk slowly. And I don’t know why, when I keep walking… I sweat a lot. I really hate to go out when the weather is hot.” (Mee Yee).

“You do not need to go out and expose to the sun, there is no need to spare extra time, in here (home), you can turn on the computer, at nine something or ten something. Turn it on, have the battery charged, just like that. Wait for a while, there will be some English words that I don't understand…after that the screen will pop out.” (Yuet Ho).

“It’s really good, at least sometimes those senior… um… (they) don’t walk so well, it is not convenient (for them to travel somewhere to join exercise class). it’s good that we can follow (instructions) and move a bit at home.” (Siu Ling).

Researcher felt the perception of “ease” perceived by participants. With minimal preparation, participants sat in front of tablet or computer just before the class started and once screen was connected and the voice of the instructor was heard, the quiet home environment was magically transformed to a vibrant “exercise class” environment. During the whole process, participant just calmly and familiarly navigated their home without any hassle.

### Technological adaptation

The superordinate theme was supported by three subordinate themes, “technological exploration”, “technological support”, and “technological advancement”.

“Technology” is not something this oldest-old cohort familiar with. In their tele-exercise experience, they mentioned that they went through different stages of struggles to get familiar with tele-exercise participation:

“(I am) not used to it initially, like I don’t know how to press the buttons. (So I) learn again, and try to press again… but why I can’t press it? I press wrongly or I didn’t press?” (Yuet Ho).

“At first, we don’t know anything. O! Dying! What to do? Now I keep learning, (I can) manage so so now…” (Siu Fong).

“At first, I tried to operate by myself, but not successful, (I) call them (staff from community centre) and ask.” (Siu Ling).

Although all participants struggled at the beginning, they all acknowledged the support from staff or the instructor in troubleshooting technical issues:

“We're pretty old. We don’t know how to use those smartphones. Sometimes we need to take it to the centre, ask the staff, when the phone isn’t functioning, they'll teach us how to do and how to press those buttons, which buttons we had pressed wrongly, they'll tell us.” (Siu Fong).

“Sometimes (I) asked the instructor (about the technical problem), the beginning was like that, now (I) won’t.” (Yuet Ho).

After going through the exploration and support stage, oldest-old participants experienced technological advancement in their tele-exercise experience:

“someone helped me set up the app, he told me there's an app called 'Zoom', you click the 'plus' button, add the password to it, I can directly join this exercise class, very easy, because the setting is more or less the same.” (Mei Ling).

Yuet Ho mentioned the concept of learning new things:

“I think I have learnt one more thing, we keep learning as we grow old, if I (do not) participate this online exercise, you (i) don't know what is computer, and (I) don’t know how does the computer look like, the computer now is so thin...learn one more thing.”

All participants talked about the technological challenges they had at the beginning, but researcher’s observations revealed that all participants could turn on the tablet or computer, occasionally hiccups such as mistyping password or mis-clicking hyper link occurred, but they all successfully joined the tele-exercise class by themselves without any help from third person during researchers’ home visit.

## Discussion

The study illustrates the first attempt to explore the lived experience of tele-exercise among the oldest-old in the tele-health era. The superordinate themes highlight four key findings. First, the ambivalent perception of safety in their experiences. Second, they appreciated that they are guided and reminded to move their aged body. Third, the overall lived experience of “ease” in their home-based tele-exercise training. Forth, the on-going technological adaptations in their experience.

The oldest-old women in this study perceived their homes as the safest place to exercise. They felt reassured by the exercise intensity prescribed by the instructor and appreciated the autonomy to adjust exercises according to their health conditions. The theme of perceiving home as the safest place to exercise was not found in previous tele-exercise qualitative study, in which the majority of participants were younger older adults [21]. Previous literature which suggested the importance of having home-based exercise intervention for the oldest-old individuals mainly focused on the reduction of transportation barrier [8], but not the strong sense of safety and security feeling at home [10]. The finding of compromised supervision quality was similar to findings from previous tele-exercise studies among adults and the general older adults [12, 21]. This highlighted the importance of improving supervision quality in tele-exercise. This can be achieved by reassuring a good set up of camera angle at home that can capture all the body parts of the participants [15], and instructor giving regular feedback to participants [31].

Oldest-old women in the current study appreciated that they are being reminded and guided to move their aged bodies in their tele-exercise experience. A prior oldest-old’s phenomenological study revealed that bodily activity was connected to the feeling of being alive and maintaining independence [28]. Longitudinal quantitative data also suggested oldest-old individuals who maintained PA could achieve longer life expectancy [50]. It is critically important to facilitate the oldest-old, who face restricted mobility and a strong desire to maintain independence at home in staying active and achieving optimal well-being. Previous exercise interventions for oldest-old documented in the literature only have a home practice component without real-time direct supervision [4, 8], the finding of this study provided evidence that oldest-old appreciated receiving guidance at their own home. This finding also provided preliminary evidence tele-exercise that allow supervision remotely may improve adherence to exercise programme which as a home component [39].

Similar to what was experienced by the general younger older adults’ cohorts in previous study [21], the oldest-old women from this study found ease in the regular tele-exercise participation. Transportation to exercise facilities has been consistently identified as barriers to exercise participation and maintenance among the oldest-old [4, 8]. This study found that oldest-old experienced a sense of “ease” in participating, as they no longer needed to travel to facilities but still received guided exercise training. The ease of participation also enables them to participate regularly which is an important finding in exercise intervention research focused on the oldest-old population. The finding suggested that tele-exercise, as a new form of delivery mode, has the potential to be a preferred delivery mode among the oldest-old as it reduces their key exercise barrier due to old age.

Oldest-old women of this study experienced technological exploration, support and advancement in their tele-exercise experience. Research has been suggesting an evolving partnership between technology and aging [27]. Accumulating evidences suggests that technology-enhanced interventions (eg., e-health, mHealth or activity trackers) are useful in promoting PA or lifestyle change among old adults, both with and without chronic diseases [16, 32, 40, 46]. A review also found that technology-enhanced home-based exercises were associated with positive health outcomes in older adults [7]. Yet, there has been a lack of understanding regarding how the oldest-old adults experience technology-enhanced health interventions. In the current study, the oldest-old participants experienced a process of technological adaptation. They faced challenges, explored, and continuously adapted to the technological aspects of tele-exercise. The finding also highlighted the crucial role of support from administrative or technical staff and the instructor in their adaptation process. These findings align with previous tele-exercise study conducted among older adults which investigated a wider spectrum of ages, from 55 to 89 years [21]. It indicates that some oldest-old women could also adequate well to technology-enhanced interventions. These findings suggest that with adequate support, the oldest-old population can have tele-exercise experiences similar to their younger counterparts.

### Limitations

The current study had several limitations. First, the study adopted repeated interviews and observations conducted through home visits. Although there was an observation component, the investigation of lived experience heavily relies on the verbal and communication abilities of the interviewees. The depth of the investigation was inevitably affected by the verbal and communication abilities of interviewees. Second, all the interviewees were female, which raises uncertainty about the experiences of male oldest-old individuals. Third, the home observation component of the study excluded individuals who had prior tele-exercise experience but discontinued participation at the time of the study, this may have excluded those with negative tele-exercise experiences from the study and introduced a potential bias. Forth, the voluntary nature of participation in the study may have attracted oldest-old women who had more positive experiences, potentially introducing a bias in the findings.

### Strengths

Although reaching the oldest-old group is not difficult, finding oldest-old individuals who have tele-exercise experience and are willing to participate in the current study is extremely challenging. This study is the first to investigate the lived experience of tele-exercise in great depth among an important subgroup of community-dwelling older adults (i.e., the oldest-old). The first strength lies in the use of repeated interviews, home visits and observations to triangulate and improve the creditability of the data. The second strength is the community-wide recruitment that ensured that participants were not limited to a single service provider for tele-exercise. This study offers valuable insights into enhancing the delivery of tele-exercise for this important and homebound sub-group in the future.

## Conclusion

The current study provides valuable insights into the role of tele-exercise plays in assisting the oldest-old in participating and maintaining their PA levels at home, especially in the emerging tele-health era. Tele-exercise offers an alternative for the oldest-old to participate in exercise classes from the comfort of their homes, where they felt more at ease in engaging and maintaining their exercise routines. They appreciated to be guided and reminded to move their aged body at home, an environment that they felt the safest. At the same time, they had ambivalent feelings about the quality of supervision, and they kept adapting to the technical challenges in their experience.

### Practical and theoretical implications

All six oldest-old women welcomed this new form of exercise delivery. In Hong Kong, the oldest-old population typically has fewer exercise class options than their younger counterparts, as traditional community centres mainly offer in-person exercise classes for those who are relatively healthy and can travel independently. With advances in telecommunication technologies and tele-exercise delivery, our study findings serve as a starting point in understanding the potential of this new delivery mode in assisting the oldest-old to maintain optimal levels of wellness and aging in place. Our results provided directions on how tele-exercise interventions should be tailored for this age group. A pilot randomized controlled trial should be conducted to investigate feasibility, acceptability, and preliminary effectiveness of this intervention, and to explore the transferability of the results to different cultural, socioeconomic, and geographical settings.

## Supplementary Information

Supplementary Material 1.

Supplementary Material 2.

## Data Availability

Data analyzed in the current study are available from the corresponding author on reasonable request.

## Abbreviations

PA: Physical Activity
IPA: Interpretative phenomenological approach
