# Supplementary material for: Vitality at home: a phenomenological study of tele-exercise in women aged 80 and older
Source: Eur Rev Aging Phys Act. 2024 Sep 19;21:25. doi: 10.1186/s11556-024-00360-9 (PMC11412020; doi:10.1186/s11556-024-00360-9)
Supplement: Supplementary file 1 — Supplementary Material 1. [file 11556_2024_360_MOESM1_ESM.docx]

Additional file 1

Video footage from home visits

<https://youtu.be/pLw9_PO9H5o>
